# S‐Equol Alleviates Palmitic Acid–Induced Muscle Atrophy by Modulating the ERβ–AMPK–Autophagy Axis in L6 Cells

**DOI:** 10.1155/jnme/9719495

**Published:** 2026-09-23

**Authors:** Shengcai Yu, Xiangmin Ni, Haoyu Li, Heng Lu, Meng ran Shi, Wenyi Zhu, Rong Fan, Xinyu Liang, Guiming Zhang, Jian Wang

**Affiliations:** ^1^ Department of Nutrition, The Second Affiliated Hospital, Army Military Medical University, Chongqing, China; ^2^ Hematopoietic Acute Radiation Syndrome Medical and Pharmaceutical Basic Research Innovation Center, Ministry of Education of the People’s Republic of China, Chongqing, China, moe.edu.cn

**Keywords:** AMPK, autophagy, estrogen receptor β, myotube atrophy, S-equol

## Abstract

**Background:**

Diabetic sarcopenia arises from metabolic imbalance–induced ROS accumulation and oxidative stress, which exacerbate muscle degeneration. Typically, autophagy removes excessive ROS and damaged organelles, helping to limit oxidative stress and preserve skeletal muscle function. S‐Equol (Eq), an intestinal metabolite of soy isoflavones with high affinity for estrogen receptor *β* (ERβ), exhibits estrogen‐like activity, yet its role in diabetic muscle atrophy remains unclear.

**Objectives:**

This study investigated the effects of Eq on diabetic muscle atrophy in PA‐induced atrophic L6 myotubes.

**Methods:**

Here, palmitic acid (PA)–treated L6 myotubes were used to model diabetes‐associated atrophy. Then, Eq treatment was further carried out, and oxidative stress, mitochondrial morphology, and autophagy were further detected.

**Results:**

PA suppressed AMPK/ULK1‐mediated autophagy, induced ROS accumulation, disrupted mitochondrial morphology, and caused myotube atrophy. Eq treatment restored autophagic activity, significantly decreased ROS and malondialdehyde levels, maintained mitochondrial integrity and function, and preserved myotube structure. Importantly, inhibition of autophagy with 3‐MA abolished the protective effects of Eq, confirming that its antiatrophic actions are autophagy‐dependent. Mechanistically, knockdown of ERβ or AMPK prevented Eq from activating autophagy, suggesting that the ERβ–AMPK signaling axis is involved in Eq‐mediated autophagy regulation.

**Conclusions:**

Our findings demonstrate that Eq alleviates PA‐induced myotube atrophy by promoting autophagy, potentially through modulation of the ERβ–AMPK signaling axis. These results highlight Eq as a potential nutritional therapy for the prevention and treatment of diabetic sarcopenia.

## 1. Introduction

Sarcopenia is characterized by the progressive and accelerated loss of skeletal muscle mass and function, leading to an increased risk of falls, fractures, physical disability, reduced functional capacity, diminished quality of life, and higher mortality [1]. Globally, approximately 10%–27% of individuals are affected by sarcopenia. Multiple factors contribute to its onset, including aging, malnutrition, physical inactivity, and various comorbidities. Among disease‐related causes, diabetes mellitus has been recognized as an important independent risk factor for sarcopenia [2]. Currently, there are about 596 million people worldwide living with diabetes, accounting for nearly one‐ninth of the global population, making it the third most prevalent noncommunicable disease. In patients with Type 2 diabetes mellitus (T2DM), notable alterations in body composition occur, characterized by increased visceral fat deposition alongside reduced muscle and bone mass. Importantly, skeletal muscle is responsible for nearly 80% of postprandial glucose utilization [3], and insulin resistance in skeletal muscle is a hallmark of T2DM. Disruptions in glucose and lipid metabolism are regarded as major contributors to the development of sarcopenia in T2DM patients [4].

As a critical energy substrate, impaired glucose metabolism in diabetes results in insufficient energy supply, which accelerates fat breakdown as a compensatory mechanism. The released free fatty acids are transported via the bloodstream to the liver and skeletal muscle for *β*‐oxidation, thereby providing additional energy. However, the accumulation of long‐chain fatty acids, particularly palmitic acid (PA), elevates reactive oxygen species (ROS) levels in skeletal muscle, exacerbating oxidative stress in diabetic individuals [5]. In parallel, excess free fatty acids in skeletal muscle are often accompanied by mitochondrial damage, which not only impairs the clearance of ROS but also promotes further ROS production from dysfunctional mitochondria [6]. Notably, oxidative stress has been implicated in the pathogenesis of sarcopenia [7], suggesting that effective ROS scavenging and attenuation of oxidative stress may represent a promising therapeutic strategy for diabetes‐associated sarcopenia.

Autophagy is a critical cellular process that depends on lysosomes to degrade transported substrates, thereby facilitating self‐repair and self‐renewal. It plays an essential role in clearing damaged organelles and maintaining cellular homeostasis [8]. Moreover, autophagy contributes to the removal of excessive ROS and the regulation of oxidative stress. Studies have suggested that impaired autophagy reduces the cellular capacity to cope with oxidative stress under various pathological conditions, including sarcopenia [9]. In addition, autophagy eliminates damaged mitochondria, preserves mitochondrial morphology and function, and mitigates oxidative stress levels. Mechanistically, under external stimuli such as nutrient deprivation, the AMP‐activated protein kinase (AMPK) signaling pathway is activated, which subsequently stimulates its downstream target UNC‐51‐like kinase 1 (ULK1). This activation promotes the assembly of the ULK1 complex and initiates autophagy [10]. Notably, AMPK also enhances the expression of Mitochondrial fusion–related protein mitofusin 2 (MFN2) and Dynamin‐related protein 1 (DRP1), thereby contributing to mitochondrial functional recovery [11]. Furthermore, in transgenic mouse models with defective autophagy, elevated oxidative stress and mitochondrial dysfunction have been shown to aggravate muscle degeneration and atrophy [12].

Previous studies have shown that premenopausal women have a lower risk of developing diabetes and cardiovascular complications compared to postmenopausal women and men, suggesting a protective role of estrogen against diabetes [13]. In addition, estrogen deficiency in postmenopausal women has been reported to increase the risk of muscle atrophy [14]. Notably, estrogen can activate AMPK, promote autophagy, and maintain mitochondrial homeostasis [15], which may mechanistically explain its protective effects on skeletal muscle function. S‐Equol (Eq), one of the characteristic metabolites of soy isoflavones in the intestine, shares a high structural similarity with estradiol and exhibits strong binding affinity to estrogen receptor *β* (ERβ). Compared to its precursor compounds, S‐Eq demonstrates superior estrogen‐like biological activity. Studies have reported that S‐Eq exerts multiple beneficial effects, including alleviating inflammation [16], protecting cognitive function [17], promoting glucose utilization [18], and ameliorating dyslipidemia [19]. However, whether S‐Eq can improve sarcopenia—particularly diabetes‐associated sarcopenia—by preventing muscle loss and maintaining metabolic homeostasis remains unclear, and the underlying mechanisms require further investigation.

In this study, PA‐treated L6 myotubes were employed to mimic muscle atrophy under diabetic conditions, and the potential therapeutic effects of Eq were investigated. Given that autophagy exerts protective effects against sarcopenia by reducing ROS accumulation and mitigating mitochondrial damage, we further examined the influence of Eq on these biological processes. Mechanistically, by silencing ERβ and key autophagy–related proteins downstream of AMPK in skeletal muscle cells, we sought to verify whether Eq alleviates muscle atrophy under diabetic conditions through the ERβ/AMPK‐mediated regulation of autophagy. The results of this work are expected to provide an experimental foundation for the prevention and treatment of sarcopenia in patients with Type 2 diabetes.

## 2. Materials and Methods

### 2.1. Materials

Rat L6 myoblasts were obtained from the Nutrition and Food Safety Research Center of the Army Medical University. S‐Eq was purchased from Daicel Chiral Technologies (China). Other reagents included Dulbecco’s modified Eagle medium (DMEM) high‐glucose medium, fetal bovine serum (FBS), and trypsin‐EDTA solution (Procell, China); PA (Xilong Scientific Co., China); glucose assay kit (Nanjing Jiengcheng Bioengineering Institute, China); PAGE Gel rapid preparation kit (Mishushengwu, China); hypersensitive enhanced chemiluminescence (ECL) kit; CCK‐8 kit; RIPA lysate; BCA protein assay kit; malondialdehyde (MDA) detection kit; mitochondrial red fluorescent probe; Myosin Heavy Chain (MyHC) antibody (Beyotime, China); p62, MFN2, DRP1, LC3B, p‐AMPK, AMPK, p‐ULK1, and ULK1 antibodies (Abmart, China); hematoxylin and eosin (HE) staining solutions; GAPDH and HRP‐conjugated secondary antibodies (Sangon Biotech, China); 3‐methyladenine (3‐MA) (Abclonal, China); LC3B lentiviral transfection kit; siRNA‐AMPK (Xuan Zun Bioscience, China); and siRNA‐ERβ (Gen Pharma, China).

### 2.2. Cell Culture

L6 cells were cultured in a growth medium containing DMEM with high glucose, 10% FBS, and 1% penicillin–streptomycin at 37°C in a humidified incubator with 5% CO_2_. When the cells grew to approximately 80% confluency, the medium was replaced with DMEM containing 1% FBS to induce differentiation. The cells were cultured for 5–7 days to allow myotube formation, which were subsequently used for experiments.

### 2.3. Cell Viability Assay

L6 cells were seeded in 96‐well plates and treated with gradient concentrations of Eq (0, 0.1, 1, 10, and 50 μM) and 3‐MA (0, 0.01, 0.1, 1, and 10 mM) for 24 h. Following treatment, 10 μL of CCK‐8 reagent was added to each well and incubated for 30 min at 37°C. Absorbance was measured using a microplate reader, and cell viability was calculated relative to the untreated control.

### 2.4. Glucose Consumption Assay

Differentiated L6 myotubes were divided into four groups: control; PA group: 0.25 mM PA; Eq group: 0.25 mM PA + 1 μM Eq; and 3‐MA group: 0.25 mM PA + 1 μM Eq + 1 mM 3‐MA. At 0, 3, 6, 9, 12, and 24 h after treatment, the glucose levels were measured using a glucose assay kit according to the manufacturer’s instructions using the glucose oxidase–peroxidase method. In brief, culture media from each group were collected and centrifuged, and the supernatants were harvested for analysis. After adding the working solution, the samples were incubated at 37°C for 10 min, and the absorbance was measured at 505 nm. The glucose concentrations were calculated according to the standard curve. Glucose consumption was calculated based on the absorbance values relative to the standard sample.

### 2.5. ROS Detection

After interventions, L6 myotubes were incubated with an ROS‐sensitive fluorescent probe for 30 min in the dark. Cells were then washed twice with PBS, resuspended in fresh medium. The cell suspension was gently pipetted to ensure uniform distribution, and an appropriate amount of cell suspension was transferred onto glass slides and covered with coverslips. Excess cell suspension around the coverslips was carefully removed using filter paper. Fluorescence images were captured using a fluorescence microscope with an excitation wavelength of 488 nm. The fluorescence intensity of ROS was quantified using ImageJ software.

### 2.6. MDA Detection

Cells were collected and homogenized in PBS. Following centrifugation, supernatants were used to measure protein concentrations and MDA levels using the MDA detection kit as per manufacturer’s instructions. In brief, an appropriate amount of thiobarbituric acid (TBA) solution was added to the cell homogenates, followed by incubation in a 100°C water bath for 45 min. After centrifugation, the absorbance of the supernatants was measured at 532 nm. The MDA content was calculated based on the standard curve and expressed as mmol/mg protein.

### 2.7. Mitochondrial Membrane Potential

Cells were stained using a mitochondrial membrane potential detection kit. Following a 30‐min incubation, cells were washed twice with JC‐1 staining buffer, resuspended in medium, and plated onto slides. Coverslips were applied, and images were captured using a fluorescence microscope. The fluorescence intensity was subsequently quantified using ImageJ software.

### 2.8. HE Staining

Differentiated cells were treated with PA, Eq at various concentrations. Following interventions, cells were washed, fixed in 4% paraformaldehyde (10 min), permeabilized with 0.5% Triton X‐100 (15 min), stained with HE (1 min), rinsed, and imaged under a microscope.

### 2.9. Mitochondrial Labeling

Differentiated L6 cells were seeded at 1 × 10^4^/mL in glass‐bottom dishes. After 48 h adhesion, cells were stained with mitochondrial red fluorescent probe, fixed with 4% paraformaldehyde (10 min), and observed using confocal laser microscopy.

### 2.10. Transmission Electron Microscopy

L6 myotubes were harvested by enzymatic digestion and centrifuged at 1000 rpm for 5 min. The cell pellets were immediately fixed in 2.5% glutaraldehyde at 4°C overnight, postfixed in 1% osmium tetroxide, and dehydrated through a graded ethanol series. Samples were then embedded in epoxy resin, sectioned into ultrathin slices, and stained with uranyl acetate and lead citrate. The ultrastructural features of mitochondria were examined under a transmission electron microscope, and representative images were acquired.

### 2.11. LC3B Lentiviral Transfection

L6 cells were seeded at 5 × 10^4^/mL in 6‐well plates. After adhesion, 10 μL of LC3B lentivirus and 5 μL of polybrene were added. Transfection proceeded for 24 h, and efficiency was evaluated using fluorescence microscopy. Differentiated cells were seeded in glass‐bottom dishes for 12 h, and red/green fluorescence co‐localization was imaged to assess autophagy.

### 2.12. siRNA Transfection

For transfection, a 20 μM siRNA stock solution was prepared. The transfection reagent was diluted in serum‐free DMEM at a 1:10 ratio. Subsequently, 5 μL of siRNA solution was mixed with 50 μL of the transfection reagent diluent to form the transfection complex, which was added to each well. After 8 h of incubation, the medium was replaced with DMEM containing 10% FBS. The si‐ERβ sequences were sense 5′‐GCUGAAUGCUCACACGCUUTT‐3′ and antisense 5′‐AAGCGUGUGAGCAUUCAGCTT‐3′; si‐AMPK sequences were sense 5′‐CGAGAGCAUGAAUGGUUUAAATT‐3′ and antisense 5′‐UUUAAACCAUUCAUGCUCUCGTT‐3′. Cells were cultured for 24 h posttransfection, and then treated with or without PA (0.25 mM) and Eq (1 μM) for 24 h before collection for Western blot analysis.

### 2.13. Western Blot

Total protein was extracted from L6 myotubes using RIPA lysis buffer supplemented with 1 mM protease inhibitors. Protein samples were mixed with loading buffer, separated by SDS‐PAGE, and transferred onto PVDF membranes. Membranes were blocked with 5% BSA and incubated with the appropriate primary antibodies, followed by horseradish peroxidase–conjugated secondary antibodies. Protein bands were visualized using an ECL detection system.

### 2.14. Statistical Analysis

Data were analyzed using SPSS 20.0 software. Data are presented as mean ± SEM. Comparisons among multiple groups were performed using one‐way ANOVA followed by Tukey–Kramer’s post hoc test. *p* < 0.05 was considered statistically significant.

## 3. Results

### 3.1. PA Suppressed Autophagy and Induced Myotube Atrophy

To mimic muscle atrophy under diabetic conditions, L6 cells were treated with PA for 24 h after 5 days of differentiation. Under normal conditions, myotubes gradually formed over time, accompanied by a progressive increase in MyHC protein expression (Figures 1A–C). Upon PA stimulation, HE staining showed that 0.25 mM PA significantly reduced myotube diameter without affecting myotube length (Figures 1D–F). Western blot analysis revealed that 0.25 mM PA markedly decreased MyHC protein levels compared to the 0 mM PA group (Figures 1G–H).

FIGURE 1PA suppressed autophagy and induced atrophy in L6 myotubes. (A) Morphological changes of L6 myoblasts during differentiation. (B–C) Representative immunoblots for the protein expression of MyHC. (D) HE staining of L6 myotubes for increasing concentrations of PA. (E) Myotube length. (F) Myotube diameter. (G–H) Representative immunoblots for the protein expression of MyHC (*n* = 3). (I, K) LC3B lentivirus transfection fluorescence and quantitative analysis (*n* = 5). (J, L–N) Representative immunoblots for the phosphorylation of AMPK and the expression of P62, LC3‐I, and LC3‐II (*n* = 3). (O) Fluorescence imaging of ROS after 6 h treatment (*n* = 3). (P) ROS levels at 1 h, 3 h, 6 h, 12 h, 24 h (*n* = 3). (Q) MDA level after 24 h (*n* = 6). Data are presented as means ± SEM. Statistical differences were determined by one‐way ANOVA followed by Tukey’s multiple comparisons test. ^∗^ *p* < 0.05, ^∗∗^ *p* < 0.01, and ^∗∗∗^ *p* < 0.001.

**Figure:**
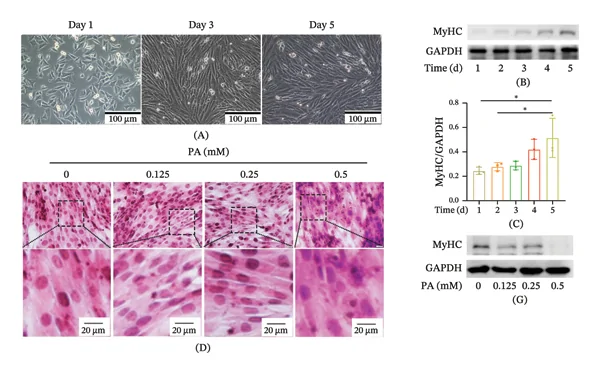

**Figure:**
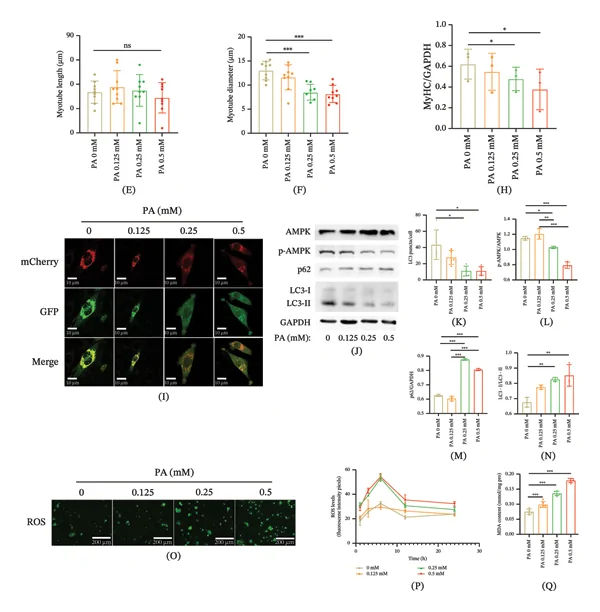

To investigate whether PA‐induced myotube atrophy is associated with autophagy inhibition, cells were transfected with an LC3 lentivirus to assess autophagy levels via red and green fluorescence colocalization. LC3B colocalization decreased in the 0.25 mM and 0.5 mM groups compared to the 0 mM and 0.125 mM groups, indicating reduced autophagy (Figures 1I and K). Moreover, Western blot analysis showed that 0.25 mM PA significantly decreased AMPK phosphorylation, increased the LC3‐I/LC3‐II ratio, and elevated p62 expression (Figures 1J, L–N). ROS fluorescence intensity and MDA content increased in a dose‐dependent manner after PA treatment (Figures 1O–Q). These results suggest that PA concentrations above 0.25 mM inhibit AMPK activation and autophagy, leading to excessive ROS accumulation, which may contribute to PA‐induced myotube atrophy in L6 cells. Therefore, 0.25 mM PA was selected for subsequent S‐Eq intervention experiments.

### 3.2. Eq Promoted Autophagy in Atrophic Myotubes

**FIGURE 2:**
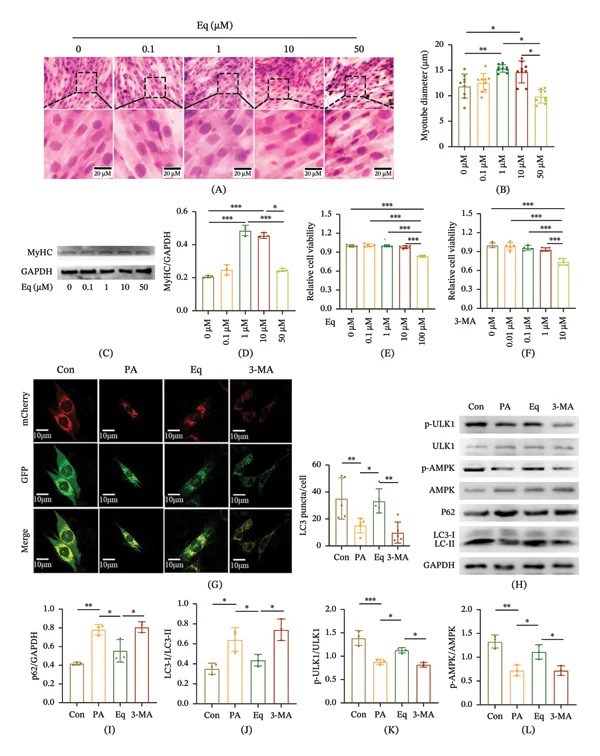
Eq promoted myotube differentiation and improved autophagy in atrophic myotubes. (A) HE staining for L6 myotubes for increasing concentrations of Eq. (B) Myotube diameter and length (*n* = 9). (C–D) Representative immunoblots for the protein expression of MyHC (*n* = 3). (E) Relative cell viability of L6 under Eq treatment (*n* = 5). (F) Relative cell viability of L6 under 3‐MA treatment. (G) LC3B lentivirus transfection fluorescence image. (H–L) Representative immunoblots for the phosphorylation of ULK1 and AMPK, and for the expression of P62, LC3‐I, and LC3‐II (*n* = 9). Con: control group with no treatment; PA: 0.25 μM PA treatment; Eq: 0.25 μM PA + 1 μM Eq treatment; 3‐MA: 0.25 μM PA + 1 μM Eq + 1 mM 3‐MA treatment. Data are presented as means ± SEM. Statistical differences were determined by one‐way ANOVA followed by Tukey’s multiple comparisons test. ^∗^ *p* < 0.05, ^∗∗^ *p* < 0.01, and ^∗∗∗^ *p* < 0.001.

L6 myotubes were treated with gradient concentrations of Eq during differentiation. Treatment with 1 μM Eq significantly increased myotube diameter and MyHC expression, indicating promotion of myotube development (Figures 2A–D). Furthermore, 1 μM Eq did not affect myotube viability (Figure 2E) and was selected for subsequent PA‐treated myotube experiments.

Considering the key role of autophagy in skeletal muscle atrophy, autophagy levels were assessed by LC3 fluorescence. PA treatment reduced red/green colocalization, whereas Eq significantly increased colocalization, indicating enhanced autophagy (Figure 2G). Western blot analysis showed that Eq increased phosphorylation of AMPK and ULK1 and decreased p62 expression and the LC3‐I/LC3‐II ratio (Figures 2H–L). The autophagy inhibitor 1 mM 3‐MA abolished these effects without affecting cell viability (Figure 2F), confirming the autophagy‐dependent action of Eq.

### 3.3. Eq Alleviated PA‐Induced Myotube Atrophy and Oxidative Stress

**FIGURE 3:**
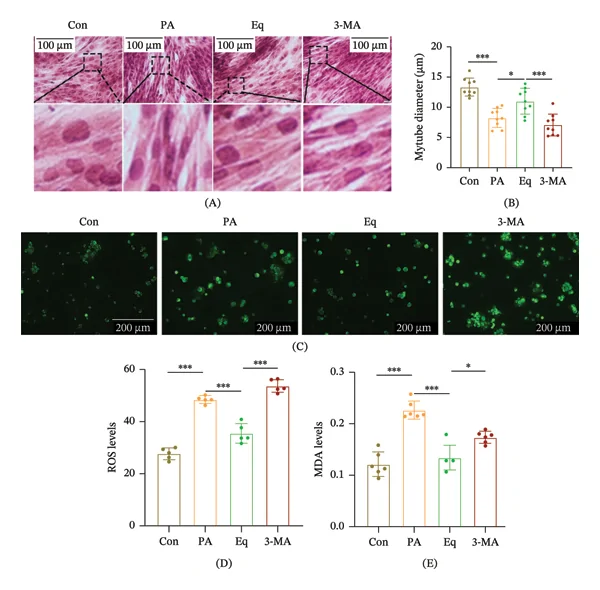
Eq alleviated PA‐induced myotube atrophy and oxidative stress. (A) HE staining of L6 myotubes for different treatments. (B) Myotube diameter and length (*n* = 9). (C–D) Fluorescence imaging of ROS after 6 h of treatment (*n* = 5). (E) MDA levels (*n* = 9). Con: control group with no treatment; PA: 0.25 μM PA treatment; Eq: 0.25 μM PA + 1 μM Eq treatment; 3‐MA: 0.25 μM PA + 1 μM Eq + 1 mM 3‐MA treatment. Data are presented as means ± SEM. Statistical differences were determined by one‐way ANOVA followed by Tukey’s multiple comparisons test. ^∗^ *p* < 0.05, ^∗∗^ *p* < 0.01, and ^∗∗∗^ *p* < 0.001.

Myotube diameter and MyHC protein expression were evaluated to examine the effect of Eq on PA‐induced atrophy. PA reduced myotube diameter, both of which were restored by Eq treatment. Autophagy inhibition abolished these beneficial effects (Figures 3A–B).

Oxidative stress was assessed by ROS fluorescence and MDA content. PA significantly increased ROS and MDA levels, whereas Eq intervention markedly reduced them. These effects were abolished under 3‐MA treatment, indicating that Eq alleviates PA‐induced oxidative stress in an autophagy‐dependent manner (Figures 3C–E).

### 3.4. Eq Improved PA‐Induced Mitochondrial Dysfunction

**FIGURE 4:**
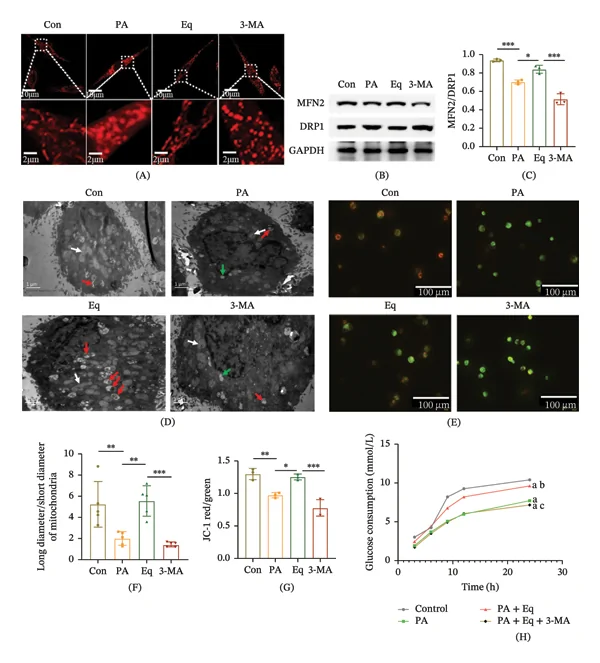
Eq improved PA‐induced mitochondrial dysfunction. (A) Mitochondrial membrane potential detection and mitochondrial probe fluorescence images. (B–C) Representative immunoblots for the protein expression of MFN2 and DRP1 (*n* = 3). (D) Transmission electron microscopy images (× 12,000); red arrows: autophagosomes, white arrows: mitochondria, and green arrows: autolysosomes. (E, G) JC‐1 detection of mitochondrial membrane potential changes (*n* = 5). (F) Mitochondrial long/short diameter ratio (*n* = 5). (H) Glucose consumption curves over time (*n* = 5). Con: control group with no treatment; PA: 0.25 μM PA treatment; Eq: 0.25 μM PA + 1 μM Eq treatment; 3‐MA: 0.25 μM PA + 1 μM Eq + 1 mM 3‐MA treatment. Data are presented as means ± SEM. Statistical differences were determined by one‐way ANOVA followed by Tukey’s multiple comparisons test. ^∗^ *p* < 0.05, ^∗∗^ *p* < 0.01, and ^∗∗∗^ *p* < 0.001. For (H): a, *p* < 0.05 vs. Con group; b, *p* < 0.05 vs. PA group; c, *p* < 0.05 vs. Eq group.

ROS accumulation is a major contributor to diabetes‐associated sarcopenia, and mitochondrial damage impairs ROS clearance, exacerbating oxidative stress. Confocal imaging showed elongated, rod‐like mitochondria in control and Eq‐treated groups, whereas PA promoted mitochondrial fission and reduced fusion (Figure 4A). The MFN2/DRP1 ratio decreased after PA treatment and was restored by Eq (Figures 4B–C). Transmission electron microscopy revealed that PA caused mitochondria to become rounded, fewer in number, and structurally damaged, whereas Eq preserved mitochondrial elongation, abundance, and structural integrity, with increased autophagosomes (Figure 4D). PA reduced the mitochondrial length‐to‐width ratio and membrane potential, both of which were recovered by Eq, and these effects were abolished by autophagy inhibition (Figures 4E–G). Furthermore, PA decreased glucose consumption, which was improved by Eq treatment; this effect was also lost under 3‐MA (Figure 4H). Collectively, these results indicate that Eq mitigates PA‐induced mitochondrial dysfunction and glucose dysregulation via autophagy, contributing to myotube recovery.

### 3.5. Knockdown of ERβ Inhibited the Effects of Eq on Promoting Autophagy in L6 Cells

S‐Eq has higher affinity for ERβ than ERα. In L6 myotubes, Eq increased ERβ expression without altering ERα (Figures S1A–C), suggesting that Eq acts primarily via ERβ. To examine the role of ERβ in autophagy, ERβ and AMPK were individually knocked down using siRNA. In control siRNA cells, PA inhibited AMPK/ULK1 phosphorylation, increased p62 expression, and elevated the LC3‐I/LC3‐II ratio, which were alleviated by Eq, indicating restoration of autophagy. In ERβ‐knockdown cells, Eq failed to restore AMPK/ULK1 phosphorylation, p62 expression, or LC3‐I/LC3‐II ratio (Figures 5A–E). Similarly, AMPK knockdown prevented Eq‐induced changes in these markers (Figures 5F–J). Notably, ERβ knockdown alone suppressed autophagy‐related protein expression, confirmed by LC3 fluorescence (Figures 5K–L), highlighting the dominant role of ERβ in autophagy. These findings suggest that Eq enhances PA‐induced autophagy by activating the AMPK/ULK1 pathway via ERβ.

FIGURE 5Knockdown of ERβ and AMPK inhibited the autophagy‐regulating effect of Eq. (A–E) Representative immunoblots for the phosphorylation of ULK1 and AMPK, and the protein expression of P62, LC3‐I, and LC3‐II in si‐NC and si‐ERβ cells (*n* = 3). (F–J) Representative immunoblots for the phosphorylation of ULK1 and AMPK, and the protein expression of P62, LC3‐I, and LC3‐II in si‐NC and si‐AMPK cells (*n* = 3). (K–L) LC3B lentivirus transfection fluorescence images and quantitative analysis (*n* = 5). Data are presented as means ± SEM. Statistical differences were determined by one‐way ANOVA followed by Tukey’s multiple comparisons test. ^∗^ *p* < 0.05, ^∗∗^ *p* < 0.01, and ^∗∗∗^ *p* < 0.001.

**Figure:**
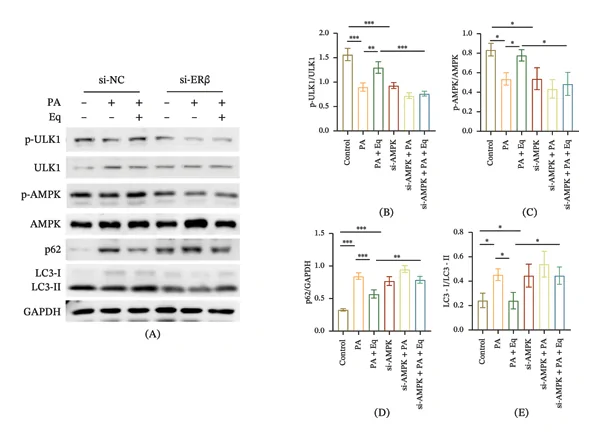

**Figure:**
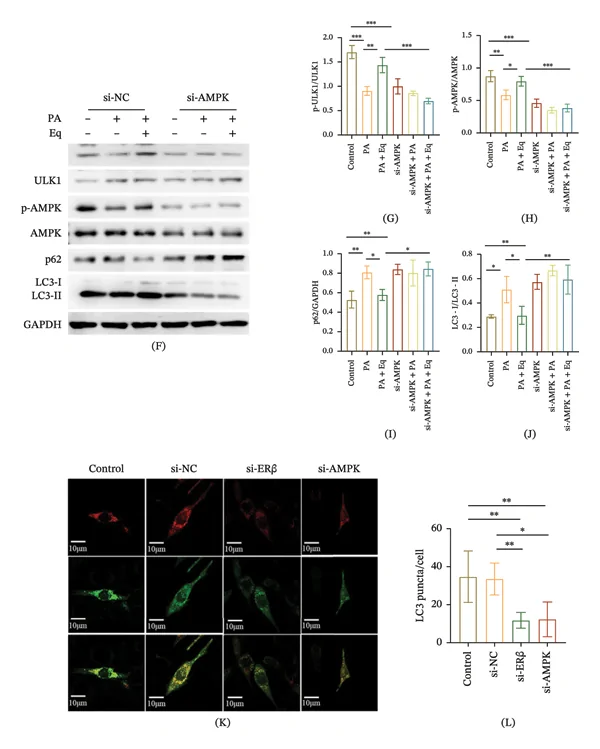

## 4. Discussion

Diabetic sarcopenia, characterized by progressive loss of skeletal muscle mass and function, is a common complication of Type 2 diabetes that impairs glucose metabolism and increases morbidity. ROS‐induced oxidative stress is a key pathogenic mechanism, and autophagy plays a crucial role in removing excess ROS and maintaining cellular homeostasis. Estrogen has been reported to promote skeletal muscle development; however, whether S‐Eq, an estrogen‐like compound, can modulate muscle atrophy in diabetic sarcopenia, and its underlying mechanisms, remain largely unknown. In the present study, PA was used to mimic a diabetic environment and induce skeletal muscle cell atrophy. Eq intervention alleviated PA‐induced myotube atrophy, reduced ROS accumulation, and restored autophagy mediated by the AMPK signaling pathway, while preserving mitochondrial morphology. Notably, treatment with the autophagy inhibitor 3‐MA significantly suppressed the effects of Eq on autophagy, ROS accumulation, mitochondrial structure, and myotube atrophy, indicating that autophagy is a key mediator of Eq’s protective action. Furthermore, knockdown experiments of ERβ and AMPK demonstrated that Eq delays muscle atrophy by regulating skeletal muscle autophagy through the ERβ/AMPK axis.

Skeletal muscle insulin resistance is a key feature of diabetes, and PA is commonly used to induce insulin resistance in myocytes to mimic a diabetic environment. In addition, 400 μM PA has been reported to activate macrophage Delta‐like ligand 4 signaling, which in turn triggers senescence in smooth muscle [20]. In C2C12 myotubes, 400 μM PA inhibited myotube formation and hypertrophy while reducing the expression of MHC IIb and IIx, isoforms specific to fast‐twitch fibers [21]. Similarly, another study showed that 250 μM PA treatment in C2C12 cells delayed myotube differentiation, promoted cellular senescence, and induced insulin resistance [22].

In the present study, L6 myotubes were treated with gradient concentrations of PA to determine the optimal concentration for inducing diabetic myocyte atrophy. Morphologically, 0.25 mM PA significantly reduced the diameter of differentiated myotubes, indicating the occurrence of atrophy. Moreover, this concentration markedly decreased MyHC protein expression, a key marker of muscle maturation. MyHC is a major structural protein of skeletal muscle, essential for resisting gravity and maintaining movement [23], and its reduction is considered a hallmark of muscle atrophy [24].

Estrogen plays an important role in regulating skeletal muscle development and maintaining normal muscle structure and function, and estrogen deficiency has been reported to induce muscle atrophy [14, 25]. As an estrogen‐like compound, treatment with 1 μM S‐Eq significantly increased MyHC expression and preserved myotube morphology, suggesting that S‐Eq exerts protective effects against muscle atrophy under diabetic conditions.

Oxidative stress is defined as an imbalance between oxidant production and antioxidant defenses, characterized by excessive ROS [26]. Elevated ROS levels have been observed in muscle atrophy models induced by nutrient deprivation or denervation [27], and antioxidant supplementation, such as glycine and N‐acetylcysteine, has been shown to reduce ROS and improve muscle strength in elderly individuals [28], highlighting the critical role of ROS regulation in muscle function. Notably, in diabetic patients, ROS levels are markedly increased due to lipid accumulation, contributing to diabetic sarcopenia. In this study, Eq intervention significantly reduced both ROS and the lipid peroxidation marker MDA, mitigating PA‐induced oxidative stress.

Skeletal muscle is rich in mitochondria, and mitochondrial damage exacerbates ROS accumulation. Mitochondrial dynamics, including fusion and fission, regulate intracellular signaling and maintain cellular homeostasis, with the MFN2/DRP1 ratio reflecting these changes and influencing glucose utilization. Under high‐energy conditions, fusion predominates with increased MFN2 expression, whereas stress conditions such as PA treatment promote fission and DRP1 expression, impairing mitochondrial glucose utilization, reducing ATP production, and decreasing MyHC synthesis. In L6 cells cultured with 22.5 mM glucose, mitochondrial fusion was enhanced, the MFN2/DRP1 ratio was high, and glucose metabolism was efficient. Eq intervention promoted autophagy, maintaining balanced mitochondrial dynamics, preserving mitochondrial structure and membrane potential, and stabilizing both metabolic and oxidative homeostasis.

Declined autophagic capacity increases the risk of muscle atrophy, as autophagy is a key mechanism for clearing ROS. A randomized controlled trial showed that elevated levels of the exercise‐induced exerkine apelin attenuated age‐related muscle atrophy by promoting autophagy in skeletal muscle [29]. In addition, acute re‐expression of TRP53INP2 in aged mice activated muscle autophagy, enhanced mitophagy, reduced ROS production, and improved muscle atrophy [30]. In the present study, PA severely suppressed autophagy in myotubes, whereas Eq maintained normal autophagic activity, contributing to lower ROS levels. Furthermore, under autophagy inhibition, the protective effects of Eq on ROS levels, mitochondrial morphology, and myotube atrophy were diminished, indicating that Eq’s regulation of diabetic muscle atrophy is autophagy‐dependent.

AMPK/ULK1 is a key signaling pathway regulating autophagy. AMPK activation accelerates the conversion of LC3‐I to LC3‐II, while p62, as a selective autophagy receptor, is degraded in autolysosomes. Previous studies have reported that exosomes from mesenchymal stromal cells ameliorate diabetes‐induced muscle atrophy by enhancing AMPK/ULK1‐mediated autophagy [31]. Similarly, in this study, PA suppressed AMPK and ULK1 phosphorylation, whereas Eq significantly restored their phosphorylation levels, accompanied by decreased LC3‐I/LC3‐II ratio and p62 expression, demonstrating that Eq promotes autophagic activity via the AMPK/ULK1 pathway.

S‐Eq and its precursor, daidzein, are structurally similar to 17β‐estradiol and selectively activate estrogen receptors ERα and ERβ. In binding assays, Eq showed stronger affinity for ERβ than ERα (Ki [ERβ] = 16 nM; *β*/*α* = 13‐fold) [32]. In this study, Eq significantly upregulated ERβ but not ERα expression in L6 cells. Daidzein has been reported to increase skeletal muscle mass in young female mice by enhancing ERβ recruitment to Ubiquitin‐specific protease 19 [33], suggesting that ERβ is a key mediator of estrogen‐like regulation of skeletal muscle growth. Estradiol can reduce ROS levels by regulating SIRT3 transcription through ERβ in human seminoma TCam‐2 cells [34], whereas tamoxifen, acting as a mitochondrial ERβ antagonist in MCF7‐BK cells, increases mitochondrial ROS.

AMPK is a critical pathway regulating autophagy, which in turn reduces ROS and maintains skeletal muscle homeostasis. In this study, Eq promoted AMPK phosphorylation and enhanced autophagy. However, ERβ knockdown abolished Eq‐induced AMPK activation and prevented improvements in LC3‐I/LC3‐II ratio and p62 levels, indicating that Eq’s regulation of autophagy depends on ERβ. Notably, ERβ can regulate autophagy via AMPK‐independent pathways, as previous studies have shown that ERβ activation promotes NLRP6‐mediated autophagy to inhibit colitis [35], and downregulates p62 to induce autophagy in osteosarcoma via mTOR inhibition [36]. In our study, AMPK knockdown also significantly reduced Eq‐mediated autophagy, demonstrating that Eq regulates ROS levels and suppresses muscle atrophy through the ERβ–AMPK‐autophagy axis.

Eq is a metabolite of soy isoflavones produced by intestinal microbiota and has been reported to exert estrogen‐like, anti‐inflammatory, and antioxidant activities [37]. As a natural source of Eq, dietary soy intake can elevate circulating Eq levels, and habitual consumption of soy or fermented soy products has been associated with a reduced risk of sarcopenia in individuals with T2DM [38]. In the present study, Eq demonstrated a protective effect against diabetic muscle atrophy by promoting ERβ–AMPK‐mediated autophagy. These findings indicate that dietary soy may serve as a practical nutritional strategy for patients with T2DM. Considering that Eq is produced exclusively from dietary isoflavones via intestinal microbial fermentation, future studies should focus on identifying and utilizing Eq‐producing bacteria to evaluate their potential as targeted nutritional interventions for diabetic sarcopenia. Furthermore, the in vitro model in the present study cannot fully recapitulate the complex systemic alterations associated with diabetic sarcopenia, including endocrine regulation, immune responses, and whole‐body metabolic interactions; further investigations using diabetic animal models are required to validate the regulation of Eq on skeletal muscle loss. In addition, Eq is relatively stable in vivo, although it can be further metabolized into several derivatives, including 3‐OH‐Eq and 6‐OH‐Eq [39]. The biological functions of these metabolites on skeletal muscle homeostasis remain to be further investigated.

## 5. Conclusions

In this study, PA‐treated L6 myotubes exhibited high levels of ROS, disrupted mitochondrial structure, and myotube atrophy, which mimicked muscle atrophy under diabetic conditions. However, Eq enhanced autophagy via the ERβ–AMPK–ULK1 pathway, which further reduced ROS accumulation, preserved mitochondrial integrity, and prevented myotube atrophy in PA‐treated myotubes. These findings underscore the potential of dietary or therapeutic Eq in preventing and treating diabetic sarcopenia, providing a strategy to improve skeletal muscle health and quality of life in T2DM patients.

**Nomenclature**
Eq: S‐Equol
T2DM: Type 2 diabetes mellitus
ROS: Reactive oxygen species
Erβ: Estrogen receptor *β*
PA: Palmitic acid
AMPK: AMP‐activated protein kinase
ULK1: UNC‐51‐like kinase 1

## Author Contributions

Yu Shengcai and Ni Xiangmin contributed equally to this work. Experimental design and manuscript writing–review and editing: Ni Xiangmin, Yu Shengcai, and Li Haoyu. Conceptualization and study supervision: Wang Jian and Ni Xiangmin. Experiment organization and implementation: Yu Shengcai, Lu Heng, and Ni Xiangmin. Experimental proponents: Shi Mengran, Zhu Wenyi, Fan Rong, Liang Xinyu, and Zhang Guiming. Visualization: Yu Shengcai. Data analysis: Yu Shengcai and Ni Xiangmin. Funding acquisition: Wang Jian and Ni Xiangmin.

## Funding

This research was funded by the Project of Chongqing Talent‐Innovative Leading Talent (Grant no. CQYC20220303514); The Young Doctor Talent Incubation Project of the Second Affiliated Hospital of the Army Medical University (Grant no. 2024YQB089); and the Open Project of Hematopoietic Acute Radiation Syndrome Medical and Pharmaceutical Basic Research Innovation Center, Ministry of Education of the People’s Republic of China (Grant no. ARSBIC‐B‐202403).

## Disclosure

All authors have read and approved the final manuscript.

## Conflicts of Interest

The authors declare no conflicts of interest.

## Supporting Information

Additional supporting information can be found online in the Supporting Information section.

## Supporting information


**Supporting Information** Supporting figure 1: Figure S1. The influence of Eq on ERα and ERβ expressions. (A) Representative immunoblots for ERα and ERβ in L6 cells under Eq treatment overtime. (B) Quantitative analysis of ER*α*. (C) Quantitative analysis of ER*β*. Data are presented as means ± SEM. Statistical differences were determined by one‐way ANOVA followed by Tukey’s multiple comparisons test. ^∗^ *p* < 0.05, ^∗∗^ *p* < 0.01, and ^∗∗∗^ *p* < 0.001.

## Data Availability

The data that support the findings of this study are available from the corresponding author upon reasonable request.
