# Supplementary material for: S‐Equol Alleviates Palmitic Acid–Induced Muscle Atrophy by Modulating the ERβ–AMPK–Autophagy Axis in L6 Cells
Source: J Nutr Metab. 2026 Sep 23;2026:9719495. doi: 10.1155/jnme/9719495 (PMC13601852; doi:10.1155/jnme/9719495)
Supplement: Supplementary file 1 [file JNME-2026-9719495-s001.docx]

Supplementary figure 1

Fig. S1. The influence of Eq on ERα and ERβ expressions. (A) Representative immunoblots for ERα and ERβ in L6 cells under Eq treatment overtime; (B) Quantitative analysis of ER α; (C) Quantitative analysis of ER β. Data are presented as means ± SEM. Statistical differences were determined by one-way ANOVA followed by Tukey’s multiple comparisons test. *P < 0.05, **P < 0.01, ***P < 0.001.
